# Demographic responses underlying eco‐evolutionary dynamics as revealed with inverse modelling

**DOI:** 10.1111/1365-2656.12966

**Published:** 2019-03-18

**Authors:** Marjolein Bruijning, Eelke Jongejans, Martin M. Turcotte

**Affiliations:** ^1^ Department of Animal Ecology and Physiology Radboud University Nijmegen The Netherlands; ^2^ Department of Biological Sciences University of Pittsburgh Pittsburgh Pennsylvania

**Keywords:** population growth rate, population model, rapid evolution, sensitivity analysis, vital rates

## Abstract

1. Changes in population dynamics due to interacting evolutionary and ecological processes are the direct result of responses in vital rates, that is stage‐specific growth, survival and fecundity. Quantifying through which vital rates population fitness is affected, instead of focusing on population trends only, can give a more mechanistic understanding of eco‐evolutionary dynamics.
2. The aim of this study was to estimate the underlying demographic rates of aphid (*Myzus persicae*) populations. We analysed unpublished stage‐structure population dynamics data of a field experiment with caged and uncaged populations in which rapid evolutionary dynamics were observed, as well as unpublished results from an individual life table experiment performed in a glasshouse.
3. Using data on changes in population abundance and stage distributions over time, we estimated transition matrices with inverse modelling techniques, in a Bayesian framework. The model used to fit across all experimental treatments included density as well as clone‐specific caging effects. We additionally used individual life table data to inform the model on survival, growth and reproduction.
4. Results suggest that clones varied considerably in vital rates, and imply trade‐offs between reproduction and survival. Responses to densities also varied between clones. Negative density dependence was found in growth and reproduction, and the presence of predators and competitors further decreased these two vital rates, while survival estimates increased. Under uncaged conditions, population growth rates of the evolving populations were increased compared to the expectation based on the pure clones.
5. Our inverse modelling approach revealed how much vital rates contributed to the eco‐evolutionary dynamics. The decomposition analysis showed that variation in population growth rates in the evolving populations was to a large extent shaped by plant size. Yet, it also revealed an impact of evolutionary changes in clonal composition. Finally, we discuss that inverse modelling is a complex problem, as multiple combinations of individual rates can result in the same dynamics. We discuss assumptions and limitations, as well as opportunities, of this approach.

## Graphical abstract

Eco‐evolutionary dynamic effects on population growth occur through effects on individual vital rates (growth, survival and reproduction). This study uses inverse modelling to estimate these vital rates for populations of aphids, showing how estimating these underlying rates provides useful insights into how ecological and evolutionary dynamics shape populations.

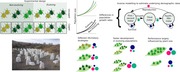

## 1 INTRODUCTION

Rapid evolution, defined as genetic changes that are fast enough to have an impact on ecological dynamics (Hairston, Ellner, Geber, Yoshida, & Fox, 2005), has been observed in a wide range of organisms (see Schoener, 2011). Given that evolutionary and ecological processes can act simultaneously, they also have the potential to interact with each other. These eco‐evolutionary dynamics potentially play an important role in shaping populations, communities and ecosystems (Bassar, Marshall, et al., 2010; Fussmann, Loreau, & Abrams, 2007; Matthews, Aebischer, Sullam, Lundsgaard‐Hansen, & Seehausen, 2016; Strauss, 2014). Discriminating between ecological and evolutionary processes and quantifying their relative importance are challenging, especially in natural populations, but different frameworks exist that aim to disentangle different processes (Coulson & Tuljapurkar, 2008; Ellner, Geber, & Hairston, 2011; Hairston et al., 2005; van Benthem et al., 2017). Experiments on eco‐evolutionary dynamics can be very useful in addition to long‐term field observations, as experiments allow for manipulating and tracking ecological and evolutionary processes (Becks, Ellner, Jones, & Hairston, 2012; Turcotte, Reznick, & Daniel Hare, 2013; Yoshida, Jones, Ellner, Fussmann, & Hairston, 2003). Experiments not only strongly test causality, but can help us understand how these processes influence each other.

Various experimental studies have now shown how density‐dependent selection can result in an eco‐evolutionary feedback loop (Strauss, 2014), both within species (Turcotte, Reznick, & Hare, 2011a; Turcotte et al., 2013) and between species (Becks et al., 2012; Yoshida et al., 2003). These, as well as other studies on eco‐evolutionary feedback loops, have often focused on population size as a response variable (Ellner et al., 2011; Hairston et al., 2005, but see Bassar et al., 2015; Cameron, O'Sullivan, Reynolds, Piertney, & Benton, 2013; Pelletier, Clutton‐Brock, Pemberton, Tuljapurkar, & Coulson, 2007). Those changes in population size however are the direct result of changes in vital rates, that is age‐ or stage‐specific survival, growth and reproduction. In other words, eco‐evolutionary dynamic effects on population growth occur through effects on vital rates.

Eco‐evolutionary studies that looked at single vital rates exist, but these have generally not considered their integrated effect on population fitness, or assumed one vital rate to be an appropriate proxy for fitness (e.g. Matthews et al., 2016). Population fitness is not equally sensitive to all vital rates (Caswell, 1978), and changes in one vital rate can be coupled with (opposite) changes in other vital rates (for instance through trade‐offs) (Stearns, 1989). Therefore, studies quantifying population fitness should ideally integrate over all vital rates (Metcalf & Pavard, 2007). For instance, Cameron et al. (2013) showed that evolution led to higher population growth rates due to increased fecundity, while survival remained unchanged. Estimating these individual vital rates gives a more mechanistic insight into the processes underlying eco‐evolutionary dynamics. Moreover, it can help us to better understand whether eco‐evolutionary dynamics operate through similar demographic mechanisms across species and systems, and determine to what extent eco‐evolutionary dynamics are repeatable.

The lack of information on the vital rates through which eco‐evolutionary dynamics operate is, at least in part, because it can be difficult to collect demographic data on individuals embedded within a population. This is especially true for the short‐lived species (e.g. zooplankton) that are typically used in multiple generation studies, as those individuals cannot easily be marked or recognized. One solution is to remove individuals from the population and measure performance on isolated individuals (Cameron et al., 2013). A drawback of this approach is, however, that density dependency in vital rates is ignored (Bassar, Lopéz‐Sepulcre, et al., 2010; Fowler, 1981). Alternatively, an interesting possibility is to use data on changes in population size and either age or stage structure over time. As those changes are the direct result of the individual vital rates, they contain information on individual survival, growth and reproduction and have been used to infer these rates. Previous studies applying this “inverse” modelling have estimated demographic rates for a broad range of different species, such as sea lions (Wielgus, Gonzalez‐Suárez, Aurioles‐Gamboa, & Gerber, 2008), blue rockfish and gopher rockfish (White et al., 2016), tropical palm species (Cropper, Holm, & Miller, 2012), the perennial plant *Cryptantha flava* (González, Martorell, & Bolker, 2016), tulip trees (Ghosh, Gelfand, & Clark, 2012) and aphids (Gross, Craig, & Hutchison, 2002). One major difficulty with the inverse estimation of individual vital rates is that many combinations of individual rates can theoretically result in the same population‐level observations (Wood, 1994). Another complicating factor is that the true underlying demographic model is unknown, which makes it challenging to decide on the functional form of the underlying vital rates, and on which covariates to include. Therefore, some prior knowledge on the biology of the system is required, for instance some demographic rates must be known beforehand (González et al., 2016).

In this study, we estimate the demographic changes in vital rates and investigate how they contribute to the eco‐evolutionary dynamics observed in the green peach aphid (*Myzus persicae*) (Turcotte, Reznick, & Hare, 2011b; Turcotte et al., 2011a, 2013). We analyse the unpublished stage‐structure population dynamics data of a field experiment (Turcotte et al., 2011a) as well as unpublished results from an individual life table experiment. In this field experiment, rapid evolution significantly altered concurrent population dynamics (Turcotte et al., 2011a). The dynamics of replicated single‐clone populations were compared to potentially evolving populations (consisting of two clones) over the course a month, approximately 3–5 generations. Rapid evolution was observed and quantified as changes in the frequency of genotypes. Rapid evolution increased exponential population growth rates by 33% to 42%, compared to non‐evolving controls, when populations were exposed to herbivores, predators and competitors. Additionally, results suggested that population density had differential fitness effects on competing clones, implying possible two‐way eco‐evolutionary dynamics between density (ecology) and evolution.

In order to gain a more mechanistic understanding of the eco‐evolutionary processes shaping the density‐dependent aphid populations, we here focus on five specific questions: (1) Which vital rates underlie the differences in intrinsic growth rate among aphid clones? (2) Can we detect trade‐offs between clones, in for instance survival and reproduction? (3) What is the impact of the changes in population density on vital rates of the three clones? (4) What demographic mechanisms evolved leading to more rapid growth in evolving populations compared to controls? Finally, (5) to what degree can we understand the evolutionary response in evolving populations, based on the vital rates of single clones?

## 2 MATERIALS AND METHODS

### 2.1 Experimental design

We used data from two different experiments. In both experiments, three aphid clonal lineages were used (which we refer to as “A,” “B” and “C”), which differ in intrinsic growth rate (Turcotte et al., 2011a). First, we used data from a field experiment on the effects of ecological context and evolution on population dynamics. The three clones were tested individually, and in each pair‐wise combination (“AB,” “BC,” “AC”), allowing for evolution (by clonal selection) to occur. At the start of the experiment, 20 third‐instar aphids (i.e. 20 individuals of one clone, or 10 individuals from each of two clones) were placed on a caged host plant (mustard; *Hirschfeldia incana*). For half of the populations, the cages were removed at day 13, allowing competitors, predators and pollinators to access the plants, resulting in a strong reduction in plant sizes compared to the caged plants (Supporting Information Appendix S1.1). In total, this resulted in 12 treatments (6 clonal treatments, fully crossed with the caging treatment), which were replicated eight times. Populations were followed over 36 days (Supporting Information Appendix S1.2). Every 3 or 4 days, the number of first/second‐, third‐, fourth/fifth‐instar and winged individuals was counted. Additionally, on these days, the number of leaves, which we used as a proxy for plant size, was counted. Plant sizes were not recorded daily; to predict daily plant size, which was implemented in the model, we used smooth functions, fitted per plant separately based on generalized additive models (Supporting Information Appendix S1.1). We excluded data from day 36, as aphid populations crashed due to plant senescence. More details on the included clones, experimental design and data collection can be found in Turcotte et al. (2011a), Turcotte et al. (2011b).

Second, we used individual aphid life table data, not published previously, which were collected during a glasshouse experiment. All aphids were maintained as clonal colonies on *H. incana* in the same glasshouse. For the experiment, on each host plant of *H. incana*, four clip cages were attached, each containing two adult female aphids. In each cage, once an offspring was born the adults were discarded. This individual was followed during its complete life and moved to a fresh leaf when leaves turned yellow. Any offspring produced were counted and removed from the cage approximately every 2 days. An average of 15.5 aphids was tested in this manner for each clone. These individual‐level data on life span, development and reproductive output were, in combination with the field experimental data, used to estimate daily survival, growth and reproduction, as explained below.

### 2.2 Modelling framework

Changes over time in the number of individuals in each stage were used to estimate demographic rates (survival, growth and reproduction). To do so, we defined three stages: (a) first/second‐instar aphids, (b) third‐instar aphids and (c) fourth/fifth‐instar and winged aphids combined. Daily changes in population structure from time *t* to time *t+*1 were described by a 3 × 3 transition matrix **A**.

(1) $$A=\left[\begin{array}{ccc} \sigma (1-\gamma ) & 0 & \phi \\ \sigma \gamma & \sigma (1-\gamma ) & 0\\ 0 & \sigma \gamma & \sigma \end{array}\right]$$

Matrix **A** describes all daily probabilities of moving from stage *i* at time *t* to stage *j* at time *t+*1, and contains three vital rates: probability of survival *σ*, probability of moving to the next stage *γ* and daily reproduction *ϕ*. Each of these three vital rates was modelled as a function of density (individuals leaf^−1^), aphid treatment and an interaction between aphid treatment and caging.

(2) $$\hat{y}=\beta_{0}+\beta_{1}D+\beta_{1+i}T_{i}+\beta_{7+i}CT_{i}$$

where *D* indicates density, and *T*_*i*_ indicates aphid treatment *i*, where *i* can vary between 1 and 6 (three single clones and three combinations of clones). *C* is a dummy variable with either 0 (caged conditions) or 1 (uncaged conditions). A total of 14 coefficients were estimated per vital rate (intercept *β*_0_, effects of density *β*_1_, aphid treatment effects *β*_2–7_ and caging effects *β*_8–13_). In the Section “*Model verification*,” we give more information on why we chose this model structure. The linear predictor $\hat{y}$ was related to the response variable by an appropriate link function. A log link function was used for reproduction ($\phi =\text{exp}\;(\hat{y})$), and a logit link function was used for survival and growth (e.g. $\sigma =1/(1+\text{exp}\;\left(-\hat{y}\right)$). The approach detailed here means that we assumed that individuals could transition only one stage per day and that all three vital rates were (linearly) affected by the same covariates.

All models were fitted in a Bayesian framework, implemented in JAGS software using the r‐package *rjags* (Supporting Information Appendix S2, Plummer, 2016). Three chains were run in parallel, and we checked convergence by Gelman and Rubin's convergence diagnostic (using 1.05 as a threshold for each parameter). We used a burn‐in period of at least 50,000 (which was extended if convergence was not yet achieved), and we took 50,000 samples from the posterior distributions after convergence. The posterior estimates were used to perform various analyses to quantify the demographic differences between the different experimental treatments, as described in “*Population‐level effects of clonal identity and evolution*.”

### 2.3 Prior distributions and likelihood

We used vague priors for all coefficients (normal distributions with mean set at 0 and precision set at 0.1). To compare population‐level observations with predictions, the likelihood was calculated in accordance with González et al. (2016). To optimize the stage distribution, we used a multinomial distribution:

(3) $$p\left(t\right)∼\;\text{Multinom}\left[\hat{N}\left(t\right),\hat{\;\;p}\left(t\right)\right]$$

Here, ***p**(t)* is a vector containing the observed proportions of individuals stage 1–3 at day *t* and $\hat{p}\left(t\right)$ are the predicted proportions. Total predicted population size is given by $\hat{N}\left(t\right)$. To compare the total estimated and observed population size, we used a Poisson distribution:

(4) $$N\left(t\right)∼\text{Pois}\left[\hat{N}\left(t\right)\right]$$

where *N(t)* is the total observed population size. Both $\hat{p}\left(t\right)$ and $\hat{N}\left(t\right)$ were predicted by the following procedure: we started with the observed population structure at the previous measurement day. Given matrix ***A***_***θ***_, calculated with parameters ***θ*** and using the functions described in Equation (2) (with the relevant link function), we projected population structure 1 day later by multiplying the observed population structure with ***A***_***θ***_:

(5) $$n\left(t+1\right)=\left[\begin{array}{c} n_{1}\left(t+1\right)\\ n_{2}\left(t+1\right)\\ n_{3}\left(t+1\right) \end{array}\right]=A_{\theta }\cdot \left[\begin{array}{c} n_{1}\left(t\right)\\ n_{2}\left(t\right)\\ n_{3}\left(t\right) \end{array}\right]$$

The resulting population structure was used to multiply with ***A***_***θ***_ again, for a total of Δ*t* times, where Δ*t* indicates the time interval between measurements (either 3 or 4 days). Finally, ***n***(*t* + Δ*t*) was divided by its sum ($\hat{N}\left(t\right)$), obtaining $\hat{p}\left(t\right)$. We recalculated ***A***_***θ***_ every time step, taking into account the population size and plant size on each day. This approach enabled us to estimate the *daily* transition matrix **A**, even though observations were on a 3‐ or 4‐day interval.

We additionally compared the observed life table data to the predicted individual survival, growth and reproduction rates. The life span of a total of 46 individuals (15 or 16 individuals for each clone A–C) was recorded during a glasshouse experiment and was on average 24 days. We calculated the predicted survival probability when density was set at 1 individual per leaf (*σ*(*D* = 1)), as this is in agreement with the life table experimental conditions. Each observed life span of individual *i* was then compared to the daily mortality probability (1 − *σ*(*D* = 1)) using an exponential distribution. For reproduction, we included daily reproduction rates for individuals from the day they started reproducing and onwards. On average, daily reproductive output of adult individuals equalled 2.2 and ranged between 0 and 7. These 635 observations on numbers of offspring were compared to the predicted reproduction when density set at 1 (*ϕ*(*D* = 1)) using a Poisson distribution. Finally, we used 45 observations on the day of maturation; on average, individuals first reproduced when they were 11.4 days old. Translating this to the population matrix shown in Equation (1), this implies that individuals reach stage 3 after on average 11.4 days. The predicted growth when density set at 1 (*γ*(*D* = 1)) was used to calculate the expected time before first reaching stage 3 (i.e. the mean first passage time), conditional on survival, as: 1+2/*γ*(*D* = 1). We compared this expected time to the observed individual maturation times using a gamma distribution, in which we estimated both the shape and rate parameter. Note that, although we used the individual life table data to estimate survival, growth and reproduction, we purposely did *not* use clone‐specific life table data to estimate effects of clonal treatment, but instead combined data for all pure clones. This was done in order to estimate the clonal treatment effects based on only the population‐level data.

### 2.4 Model verification

We performed four analyses for model verification: (a) we tested a range of models with different covariates (including stage effects, population size, plant size, population density and caging), fitted to each aphid treatment separately. Based on cross‐validation, we selected the covariate resulting in the highest predictive ability across treatments, and defined the final model structure (Equation (2)). (b) We tested our inverse modelling approach with simulated data, for which the true relationships were known. (c) We looked at the residuals of the fitted model to ensure that the model yielded unbiased predictions. Finally, (d) we reran the model six times testing a wide range of initial values to ensure that a global optimum was found. See Supporting Information Appendix S3 for more details and results.

### 2.5 Population‐level effects of clonal identity and evolution

Using the median of the posterior distributions for each estimated parameter, we projected transition matrices for each treatment, for densities ranging between 0 and the 95% quantile per caging treatment (4,274 and 2,100 individuals leaf^−1^ for the caged and uncaged conditions, respectively). Average density was 1,024 and 416 individuals leaf^−1^ for the caged and uncaged conditions, respectively. For each matrix, asymptotic population growth rate was computed, which is the dominant eigenvalue. The matrices were used for subsequent analyses.

First, we compared the three pure clones to evaluate how clonal differences in vital rates led to differences in (density‐dependent) population growth rates. To do so, we used life table response experiments (LTREs; Caswell, 1989). An LTRE decomposes differences in population growth rate into the contribution of differences in each underlying matrix element or vital rate. As we were interested in vital rate differences between treatments, we quantified the effects of vital rate differences on the differences in population growth rates. We created a matrix for the “average” clone using the average of each of the estimated clone‐specific parameters, from which we obtained asymptotic “reference” population growth rate. Here, we first applied the relevant link function for the parameters describing survival, growth and reproduction to get averages on the response scale. For each clone, we then replaced one of the averaged vital rates by the clone‐specific vital rate, and recalculated population growth rate. The difference in growth rate between the reference growth rate and the growth rate in which one of the vital rates is replaced by a clone‐specific vital rate quantifies the population‐level effects of clonal differences in each of the vital rates, at a given density. This analysis was repeated for each density, and both for the caged and for the uncaged treatments.

Second, we quantified the effects of evolution across densities, following a similar procedure. As a reference matrix, we calculated the average matrix over each combination of two clones, by using averaged vital rates, at a given density. This reflects the “expected” transition matrix, when both populations occur at a constant frequency of 50%, which represents a non‐evolving population. We then replaced one of the vital rates by the vital rate of the corresponding mixed population, and calculated the difference between the reference population growth rate and the population growth rate in which the vital rate is replaced. This was done for each of the three mixed populations, for all densities, and both for the caged and for the uncaged treatments. To quantify uncertainty in the population‐level effects of clonal differences and of evolution, the above analyses were repeated 1,000 times with coefficients randomly obtained from the posterior distributions of each parameter.

### 2.6 Predicting population dynamics in evolving populations based on pure clones

The above analyses were based on asymptotic measures of (density‐dependent) fitness, that is assuming a stabilized stage structure at a given density. We were also interested in quantifying the importance of various processes leading to differences in *transient* daily population growth rates of the evolving populations compared to the pure clone populations, using population structures observed during the experiment. The following five steps were repeated for each observed population structure of the evolving populations.

1. We projected population size one‐time interval (3 days) later based on the estimated vital rates and the observed plant size for the corresponding evolution treatment, and considered this to be the “true” reference population size prediction at *t*+3.

We then quantified to what extent we could predict these true population sizes based on:

1. The dynamics of the pure clones. We averaged vital rates and day‐specific plant sizes of the relevant pure clones and projected population size at *t*+3. We started from the same population structure and size, but implemented the average plant size, resulting in a different density. This reflects the expected dynamics of a non‐evolving population (in which both clones occur at a constant frequency of 50%), the same plant size in the evolving and non‐evolving populations, and no interactions in vital rates among clones.
2. Observed plant sizes. Population dynamics were here projected based on the non‐evolving averaged vital rates of the pure clones, but instead of using mean plant size from the pure clones, the actual observed day‐specific plant size from the evolving population was included to calculate the density at time *t*.
3. Changing clone frequencies (evolution). We no longer assumed a constant frequency, but implemented the observed genotype frequencies of both clones, for a given day (Supporting Information Appendix S1.4). We calculated average vital rates weighted by the frequency of each of the clones and used these to predict population size at *t*+3.
4. Vital rate type‐specific changes in the evolving populations. We tested for the presence of interactions among clones resulting in changed vital rates. Survival, growth and reproduction (weighted averages from the pure clones) were one by one replaced by the estimated vital rate of the evolution treatment, and again population dynamics were projected.

Steps 2–5 were evaluated one by one, using the previous step as a starting point. For all scenarios, we calculated growth rates by dividing population sizes at day *t*+3 by population size at day *t* and translated these values to daily population growth rates. We calculated the proportion of variance explained by each of the scenarios, to assess the predictability in transient population dynamics of the evolving populations.

## 3 RESULTS

### 3.1 Model selection and estimated coefficients

**Figure 1:**
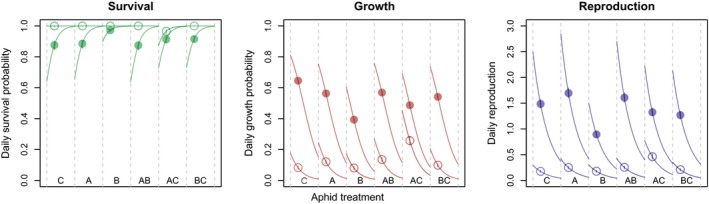
Vital rate estimates for the six aphid treatments based on the fitted model. Error bars show 95% credible intervals of the estimates due to uncertainty in aphid treatment effects. Colours indicate different vital rates (green: survival, red: growth and blue: reproduction). Dots show the estimates at average density (calculated across all observations) for the caged (closed dots) and uncaged treatments (open dots). Lines show the effects of density (aphids leaf^−1^), ranging between zero (left) and one standard deviation above the average (right)

This fitted model resulted in accurate and unbiased predictions of numbers of individuals in each stage (*r* ^2^ = 0.89). Predicted daily survival probability in the caged populations, at average density (across all observations; 815 aphids leaf^−1^), ranged between 0.87 and 0.97 and increased with density (Figure 1a; see Supporting Information Appendix S4.1 for all estimates). Survival estimates were significantly higher for clone B compared to the other aphid treatments. Average daily probabilities of moving to the next stage (growth) for caged populations ranged between 0.39 and 0.65 and decreased with density (Figure 1b). Finally, daily reproduction when caged ranged between 0.89 and 1.70 and decreased with density (Figure 1c). Clone B showed the lowest reproduction, and clone A showed a higher reproduction compared to all aphid treatments except for treatment AB.

Results suggest that both growth and reproduction were strongly decreased in the uncaged populations, in all aphid treatments (open dots in Figure 1). In contrast, survival was increased, implying a survival probability of practically 1 under uncaged conditions (Figure 1a). The estimated parameters were used for the subsequent analyses, in which we combined the vital rates to construct transition matrices (according to Equation (1)).

### 3.2 Vital rates underlying population‐level differences among pure clones

**Figure 2:**
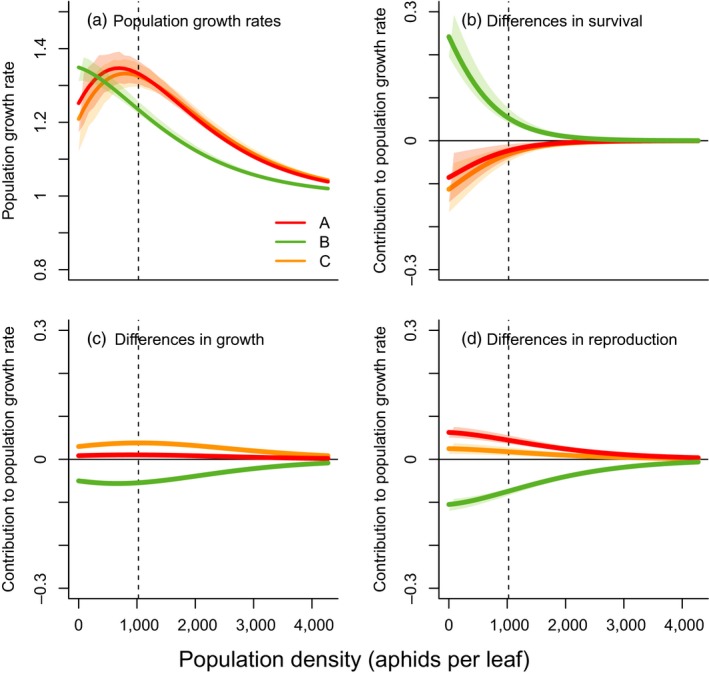
(a) Projected population growth rate as a function of density for the three pure clones, under caged conditions. (b–d) Life table response experiment comparing different clones, as a function of density (individuals per leaf). Clones A–C were compared to the average matrix across the three clones. Densities range between 0 and the 95% quantile of observed densities, under caged conditions. Different colours indicate different clones, and vertical lines indicate the average density under caged conditions. Shaded polygons show 95% confidence intervals in the predictions, obtained by simulating 1,000 transition matrices by drawing coefficients from the posterior distributions of the clonal effects

In this section, we present the results for the caged treatments (see Supporting Information Appendix S4.2 for the results of the uncaged treatments). Projected population growth rates decreased with density, after an initial increase for clones A and C (Figure 2a). Clone B had the highest population growth rate only at the lowest densities (Figure 2a), but shows the strongest negative effect of density. This results in the lowest growth rates overall. Clone A, in contrast, generally shows the highest growth rates, although the difference with clone C diminishes at higher densities. This is mostly in line with the observed trends: although clone B has higher population sizes for most of the time compared to clone C (Supporting Information Appendix S1.2), when correcting for plant size, clone B reaches lower densities (Supporting Information Appendix S1.3).

To evaluate which vital rates caused these differences in growth rates (which was our first research question formulated at the end of the introduction), we performed an LTRE. Results show that the lower population growth rate of clone B (for a given density) is caused by the lower reproduction and slower development (Figure 2b–d). This effect is partly counterbalanced by increased survival. Clone A has a slightly higher population growth rate due to a significant benefit related to reproduction (Figure 2d). These opposite patterns of growth and reproduction on the one hand and survival on the other hand might indicate trade‐offs between vital rates, as no clone benefits from increases in each vital rate (answering research question 2). These negative correlations among vital rates seem consistent, as they also appear in the mixed populations (see below).

### 3.3 Vital rates underlying population‐level evolutionary effects

**Figure 3:**
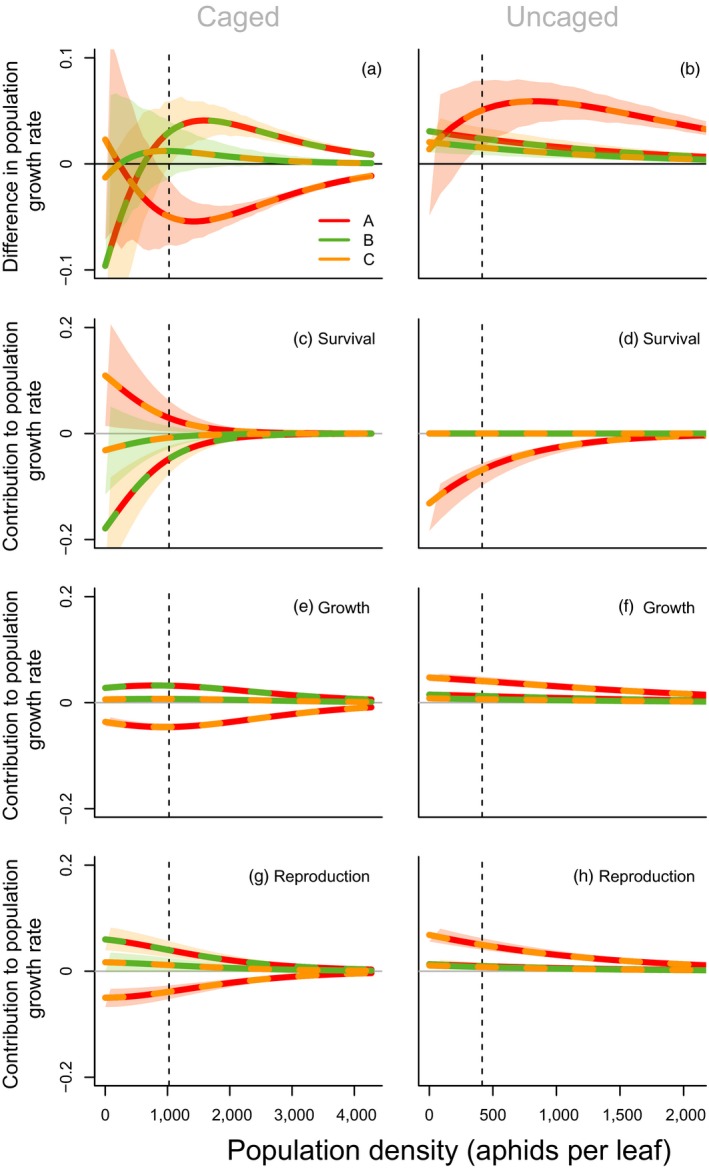
(a,b) Differences in population growth rate between the evolving population and the expected population growth rate calculated as the average of the two pure clones (i.e. at a 1:1 ratio), under caged (left) and uncaged (right) conditions. Values above zero imply that population growth rate of the evolving population is higher than expected based on the pure clones. (c–h) Life table response experiment, comparing population matrices of each mixed population to the average matrix of the two pure clones (i.e. at a 1:1 ratio): contribution of difference in c,d) survival, d–f) growth and g,h) reproduction. Positive values indicate a higher population growth rate in the evolving population due to differences in either survival, growth or reproduction. Different colours represent different combinations of pure clones. Densities range between 0 and the 95% quantile of observed densities, under caged (left) or uncaged (right) conditions. Vertical lines indicate the average density, under either caged or uncaged conditions. Shaded polygons indicate 95% confidence intervals in the predictions, obtained by simulating 1,000 transition matrices by drawing coefficients from the posterior distributions of the clonal effects

Comparing the population growth rates of the evolving populations with the expected population growth rate when both clones occur at a frequency of 0.5, complex interactions with density are found for the caged treatments (Figure 3a). In uncaged conditions, population growth rate was higher in all evolving populations across all densities (Figure 3b). In both caged and uncaged conditions, population growth rate of treatment BC is higher than the mean growth rate of B and C. The same applies for treatment AB under uncaged conditions, and at higher densities when caged. Treatment AC results in higher population growth rates than expected only in uncaged conditions. Higher growth rates for the evolving populations are mostly due to benefits related to faster development, more specifically due to higher growth (Figure 3e,f) and reproduction (Figure 3g,h) rates, in both caged and uncaged conditions. In contrast, survival of the evolving populations generally decreases population growth rates (Figure 3c,d).

### 3.4 Predicting the dynamics in the evolving populations

**Figure 4:**
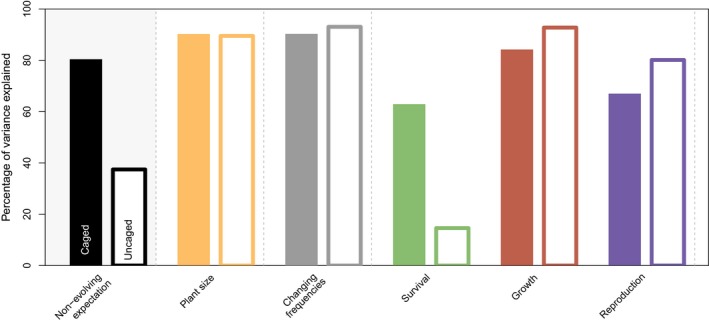
Variance explained in the transient population growth rates (over 3 days) of the evolving populations. Black bars show the explained variance when assuming a non‐evolving population, in which the vital rates equalled the average vital rates of the two pure clones. Orange bars show the explained variance when plant size is replaced by the day‐specific plant size of the mixed population. Using this as a starting point, we quantified the effect of changing frequencies, instead of assuming a constant frequency of 0.5 (grey bars). Green, red and blue bars show the proportion of variance explained when each of the averaged (weighted by the frequency) vital rates is replaced by the vital rate of the mixed population. Replacing all three vital rates at the same time results in the reference model and hence a 100% of the variance explained. For the caged populations, we used each observed population structure from day 0 until day 31; for the uncaged conditions, we used each observed population from day 17 until day 31

When assuming a non‐evolving population, in which vital rates and plant dynamics equal the 50–50 average of the two pure clones, 80% and 37% of the variance in daily population growth rates in the evolving treatments can be explained, for the caged and uncaged conditions, respectively (step 2, see “*Predicting population dynamics in evolving populations based on pure clones”* in M&M; Figure 4). For the uncaged conditions, this proportion greatly increases when including observed treatment‐specific plant sizes instead of the averaged plant size at a certain point in time (step 3; orange bars). When allowing clonal frequencies to change through time, *R* ^2^ increases from 89.5% to 93% in the uncaged conditions, but not for the caged populations (step 4; grey bars). Finally, separately replacing each of the averaged vital rates by the treatment‐specific vital rates did not improve the predictability (step 5; Figure 4). This indicates that it is a combination of changes in multiple vital rates together resulting in the dynamics of the evolving populations (replacing all three vital rates at the same time results in the reference model and hence a 100% of the variance explained). This suggests that the entire life history is evolving in the mixed populations instead of isolated vital rates.

## 4 DISCUSSION

The main goal of this study was to gain a more mechanistic understanding of the eco‐evolutionary processes shaping aphid populations, by quantifying how clones differ in individual growth, survival and reproduction and how these differences contribute to responses of evolving populations. Our density‐dependent population models show clear intraspecific variation in the degree of density dependence (Figure 2a), which is in agreement with the aphid study by Agrawal, Underwood, and Stinchcombe (2004). According to our results, clone B showed the strongest negative response to density, resulting in the competitive strength of clone B being highest only at very low densities. Clone A had the highest fitness at intermediate densities, and clones A and C are equally fit at high densities. The novelty of our study is that we additionally assessed which vital rates caused the variation in density dependence (our third research question) and which vital rates were altered in the evolving populations. Density negatively affected growth and reproduction (Figure 1), but, on a population level, interactions with density were mainly driven by survival differences between clones, with survival differences becoming smaller at higher densities (Figure 2). These genotype‐specific density effects are in accordance with experimental evidence for the presence of a full eco‐evolutionary feedback loop, as was found in a follow‐up experiment by Turcotte et al. (2013), by showing that initial density affected the rate and direction of evolution, and that evolution altered population growth.

Comparing density‐dependent population growth rates of each of the potentially evolving populations with the expected growth rate of a non‐evolving mixed population implies an effect of evolutionary dynamics on ecological dynamics, in particular under uncaged conditions (Figure 3). For all three combinations, an overall increase in population growth rate was found across all densities. Increases were due to both increased growth and reproduction, despite a decrease in survival for combination AC (answering our fourth research question). Under caged conditions, patterns were less straightforward, although for two out of three combinations, population growth rate of the evolving population was higher across most densities (Figure 3). Studies quantifying the importance of evolutionary vs. ecological factors have found varying results, with the importance of evolution practically ranging between 0% and 100% (Becks et al., 2012; Ellner et al., 2011; Govaert, Pantel, & De Meester, 2016), depending on the system and evaluated response variable. For the aphid populations under caged conditions, dynamics of the mixed populations were well predicted by assuming a non‐evolving population in which both clones occur at a 0.5 frequency, which is in agreement with the finding that evolution did not affect the growth of caged aphid populations (Turcotte et al., 2011a). In contrast, under uncaged conditions, dynamics were relatively poorly predicted when assuming a non‐evolving population. Results show that here, daily per capita growth rate was in the first place shaped by changes in plant size, as including plant size increased the explained variation in transient population growth rates from 37% to 90% (Figure 4; research question 5). It is perhaps not surprising that plant size, as a proxy for available resources, shapes population dynamics to a large extent.

We then quantified the importance of evolution, in the form of changing clonal frequencies, which had no effect under caged conditions and only a 3.5% increase in explained variance under uncaged conditions. This supports earlier findings that evolution influences population dynamics under uncaged conditions (Turcotte et al., 2011a), although the effect is very small compared to the effects of plant size. Evolution is expected to have larger impacts on population growth through time, when clonal frequencies start to deviate further from starting distributions. We would thus predict that the importance of evolution increases with time. However, given the short duration of the experiment, these temporal effects are difficult to assess, in particular for the uncaged conditions as cages were removed only at day 13. Given that the duration of the uncaged conditions was only ~2–3 generations, the 3.5% increase in explanatory power due to evolution could suggest that evolutionary dynamics potentially play an important role in shaping ecological dynamics over the longer term. Future experiments will be required to test this further.

Second, we looked at the importance of evolutionary dynamics leading to changed interactions among clones, which can also lead to increased performance, due to for instance resource partitioning or facilitation. It is widely known that grasslands with higher plant species richness show increased productivity (Tilman, Wedin, & Knops, 1996); however, also within a species, performance can increase with increasing genetic diversity (reviewed in Hughes, Inouye, Johnson, Underwood, & Vellend, 2008). For example, in springtail populations, various life‐history traits improve with genetic richness (Ellers, Rog, Braam, & Berg, 2011). We found some evidence for interactive effects on vital rates as 7% of the variation in transient growth rates remained unexplained after taking into account plant size and evolution. This suggests non‐additive effects of combined genotypes, although it could also (partly) reflect uncertainty in the estimates or perhaps changes in the composition in winged vs. unwinged adults, which both fall into the same stage in our analysis. We were unable to pinpoint a specific vital rate that explained the remaining variation all by itself (in that case, the explained variance would approach 100% when replacing one of the vital rates; Figure 4). This could suggest that these non‐additive effects of genotypic diversity do not necessarily operate through the same demographic rates, even within the same species. Future experiments will have to test this. We also note that differences in vital rates between the pure and mixed populations were generally small (Figure 1), so we might also lack the power to detect these interactive effects, if present at all.

### 4.1 Caging and density

Our findings suggest that density is the foremost important factor determining daily population growth rates (Supporting Information Table S1 in Appendix S3, Figures 2 and 3), making it critical to include plant size in the analysis (Figure 4). Results indicate negative density dependence in population growth, as was already found for these populations by Turcotte et al. (2011a), in other aphid populations (Agrawal et al., 2004; Breton & Addicott, 1992), as well as across other taxonomic groups (Fowler, 1981). With our approach, we were now able to quantify through which vital rates population growth rate decreased with density. Results suggest that this is due to reduced reproduction and growth, which is mostly in line with earlier studies, on, for example, *Daphnia* (Goser & Ratte, 1994; Guisande, 1993) and soil mites (Ozgul, Coulson, Reynolds, Cameron, & Benton, 2012). More surprising is the apparent positive relation between survival and density, which has also been observed in *Daphnia* (Bruijning, ten Berge, & Jongejans, 2018) and some developmental stages of soil mites (Ozgul et al., 2012). It could be that populations reach higher densities, because individuals survive better, leading to a positive correlation between survival and density.

We also noted a positive effect of cage removal on survival (Figure 1). Individual growth and reproduction are strongly decreased, while survival approaches 100%. While this may seem strange at first, we have two likely explanations for this pattern. First, cages were removed only at day 13 and in half of the populations. We therefore have relatively little data, from a short time period, on the uncaged dynamics (compared to the caged dynamics). During the 2 weeks of uncaged dynamics, it could—in principle—have been that almost all individuals survived. Second, higher survival in uncaged conditions might reflect the same pattern as the found positive effects of density: aphids under uncaged conditions experienced a larger (interspecific) density, due to the presence of competitors. If experienced density indeed reduces reproduction and growth but increases survival, as the estimated density effects suggest, it is perhaps not surprising that interspecific density of competitors has the same effects.

Reduced population growth rates in the uncaged populations are partly due to the smaller plant sizes. These effects are captured by the inclusion of density (population size corrected for available resources) as a covariate in the analyses. However, even after correcting for density, uncaged populations reach smaller population densities, suggesting additional effects of the cage removal (Supporting Information Appendix S1.3). Based on our results, we predict that this is not so much because of predation, as this would lead to a decrease in survival. Instead, it could be due to competition for resources by other herbivores, such as other aphid species.

### 4.2 Matrix model parameterization with inverse modelling

It has been shown that estimates of individual rates based on stage‐frequency data can be sensitive to the chosen model structure (Manly & Seyb, 1989). By first exploring which single covariate resulted in the largest model improvement, and by doing so for each clonal treatment separately, we have attempted to find the vital rate structure that is most likely to represent the true dynamics. We show that including density (number of individuals leaf^−1^) resulted in a major model improvement in most aphid treatments, suggesting a strong support for this covariate. We note, however, that we made the simplifying assumption that each vital rate is affected by the same predictors, which does not necessarily have to be the case. Moreover, we considered only one type of life cycle (Equation (1)), which seems realistic for our study species as was also confirmed by the individual life table data. Finally, we were able to inform the model on the parameters making use of the life table data, as was suggested in David, Garnier, Larédo, and Lécomte (2010).

Whether the model including effects of density, caging and treatment indeed captures the true observed dynamics is of course unknown. It could be that the model fit can be improved by including other (unknown) covariates, interactions, nonlinear effects and/or different structures for different vital rates. In addition, estimates may be sensitive to the chosen likelihood functions (Manly & Seyb, 1989), including how different components of the total likelihood are weighted (i.e. is it more important that the model yields accurate predictions of total population sizes or of population structure?). However, as the fitted model explained 89% of the variation in one‐time interval changes in stage‐specific population numbers, we are confident that we have identified the most important factors influencing dynamics of the aphid populations. Estimates of the simulations give confidence in the identifiability of the model as parameters can, in principle, be estimated accurate and unbiased (Supporting Information Appendix S3.2). We note, however, that for the simulations we used the same modelling structure as assumed in the analyses, and that the experimental data were noisier, both within and between treatments.

Moreover, although the above points make that we believe that plant size was indeed an important factor shaping the dynamics in this system, differences among clonal treatments (which was the main focus of this study) were more subtle than the effects of plant size. Indeed, most of the estimated vital rates did not differ significantly between pure clones, nor between evolving vs. non‐evolving treatments (Figure 1), possibly indicating a lack of power to detect potential differences. Whether or not the vital rate differences among clonal treatments that we did observe, indeed reflect biological differences in life‐history traits, can only be confirmed by collecting the required individual‐level data within experiments on populations.

The inverse estimation of transition matrices obviously comes with challenges, and measuring the individual rates directly (on individuals embedded in the population) is preferred. However, for small‐sized species often used in this type of experimental studies, such as zooplankton (Van Doorslaer, Stoks, Duvivier, Bednarshka, & De Meester, 2009), mites (Cameron et al., 2013) and aphids, it is difficult to follow individuals within their population. This is in contrast to studies on, for example, mammals, birds or fish (Bassar, Lopéz‐Sepulcre, et al., 2010; Grant & Grant, 2002; Pelletier et al., 2007; Traill, Schindler, & Coulson, 2014), where it is common practice to mark individuals in order to obtain demographic data. One solution is to measure individual rates on sampled individuals/genotypes, held in isolation (Cameron et al., 2013; Van Doorslaer et al., 2009). A drawback is that density‐dependent effects will be overlooked, while these are known to impact population dynamics. Alternatively, individuals can be isolated within their population to measure individual rates during a short interval (Bruijning et al., 2018). However, if these individual data are not available, we show that estimating individual rates based on stage‐frequency data can provide useful insights into how ecological and evolutionary dynamics shape populations. Moreover, our inverse modelling results in predictions on individual vital rates, which can subsequently be tested by collecting the relevant data. This will further inform us on the reliability, robustness and opportunities of inverse modelling to estimate the individual vital rates underlying changes in population dynamics.

Vital rates are not independent entities, and they often covary, positively or negatively (due to genetic correlations or trade‐offs). In addition, changes in one vital rate will affect the sensitivity of population growth to changes in all other vital rates change as well. Therefore, assessing how eco‐evolutionary effects on single vital rates affect fitness requires an incorporation of the associated vital rate changes as well. In this study, we have taken a first step by linking individual rates to population‐level responses. Eco‐evolutionary dynamics operate through individual phenotypes; however, it is largely unknown how phenotypes drive these dynamics (Rudman et al., 2018). Future studies linking vital rates to underlying phenotypes, for instance using body size‐structured population models (Bassar et al., 2015), will give a more complete picture. Ultimately, linking phenotypic traits to fitness components and their integrated effect on population fitness will greatly improve our understanding of eco‐evolutionary dynamics.

## AUTHORS' CONTRIBUTIONS

M.M.T. designed the experiments and collected the data; M.B. analysed the data; M.B. led the writing of the manuscript. All authors contributed critically to the drafts and gave final approval for publication.

## Data Availability

Data are archived in the DANS EASY repository: (https://doi.org/10.17026/dans-2ak-nnmc) (Bruijning, Jongejans, & Turcotte, 2019).
