# Supplementary material for: Demographic responses underlying eco‐evolutionary dynamics as revealed with inverse modelling
Source: J Anim Ecol. 2019 Mar 18;88(5):768–79. doi: 10.1111/1365-2656.12966 (PMC6850177; doi:10.1111/1365-2656.12966)
Supplement: Supplementary file 1 [file JANE-88-768-s001.pdf]

Supporting Information for: Demographic responses underlying eco-evolutionary dynamics as revealed with inverse modelling

Marjolein Bruijning, Eelke Jongejans, Martin M. Turcotte

Contents

S1 Observed trends **2**
S1.1 Plant sizes 2
S1.2 Population sizes 3
S1.3 Population densities 4
S1.4 Clonal frequencies 5

S2 Implementation in JAGS **6**

S3 Model verification **8**
S3.1 Testing different covariates 8
S3.2 Simulated data 9
S3.3 Residuals 10

S4 Model results **11**
S4.1 Posterior estimates 11
S4.2 LTRE for uncaged treatments 12

S1 Observed trends

S1.1 Plant sizes

To obtain densities, population numbers were divided by plant size. We used the number of leaves as a proxy for plant size. To be able to implement daily plant sizes, we fitted smooth functions, as data was collected on a three-or-four-day basis. Generalized additive models were fitted, for each plant (containing a population aphids) separately. In Fig. S1 the daily model predictions are shown. Predictions below 1 were set to 1, in order to include a minimum plant size of 1 leaf.

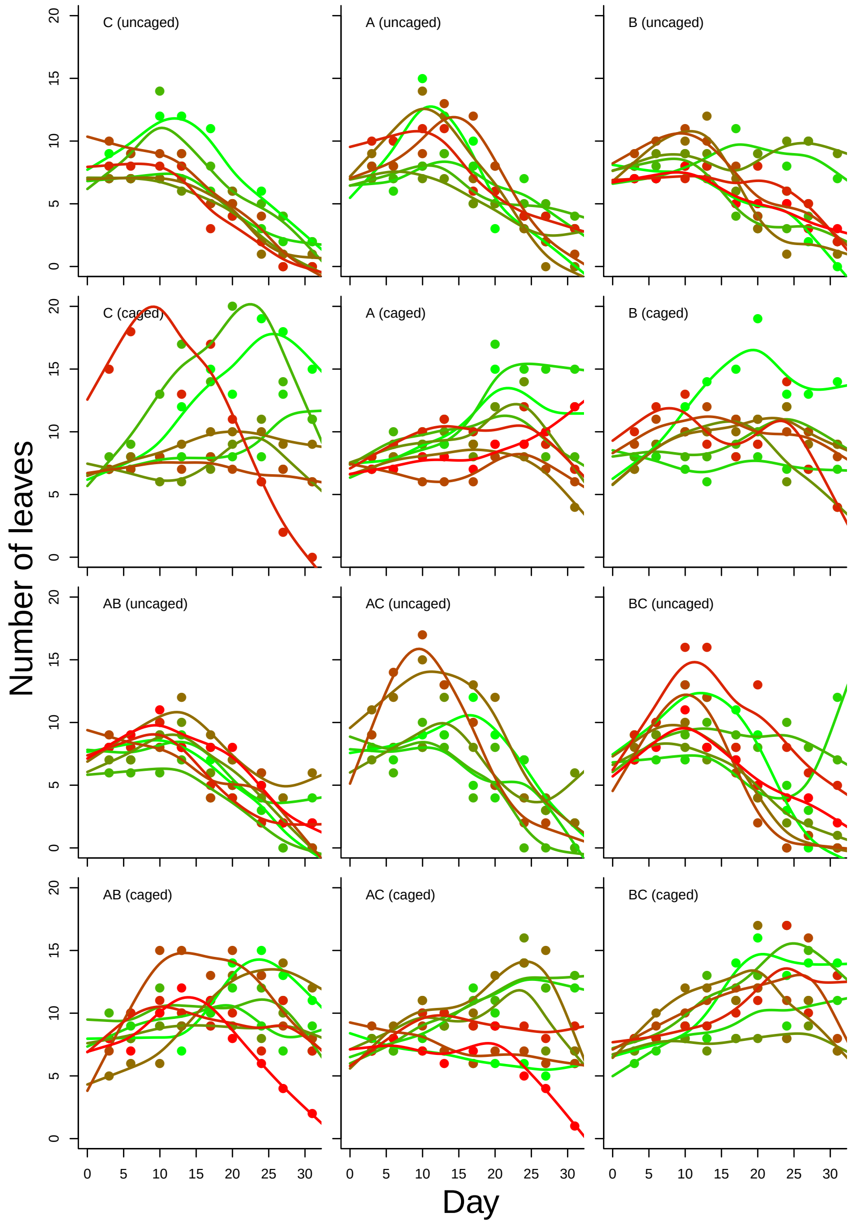

Figure S1: Changes in the number of leaves over time for each plant. Each graph shows replicates for a different aphid treatment. Different colors indicate different plants (replicates). Dots show the data and lines show model predictions based on generalized additive models, shown per experimental treatment.

S1.2 Population sizes

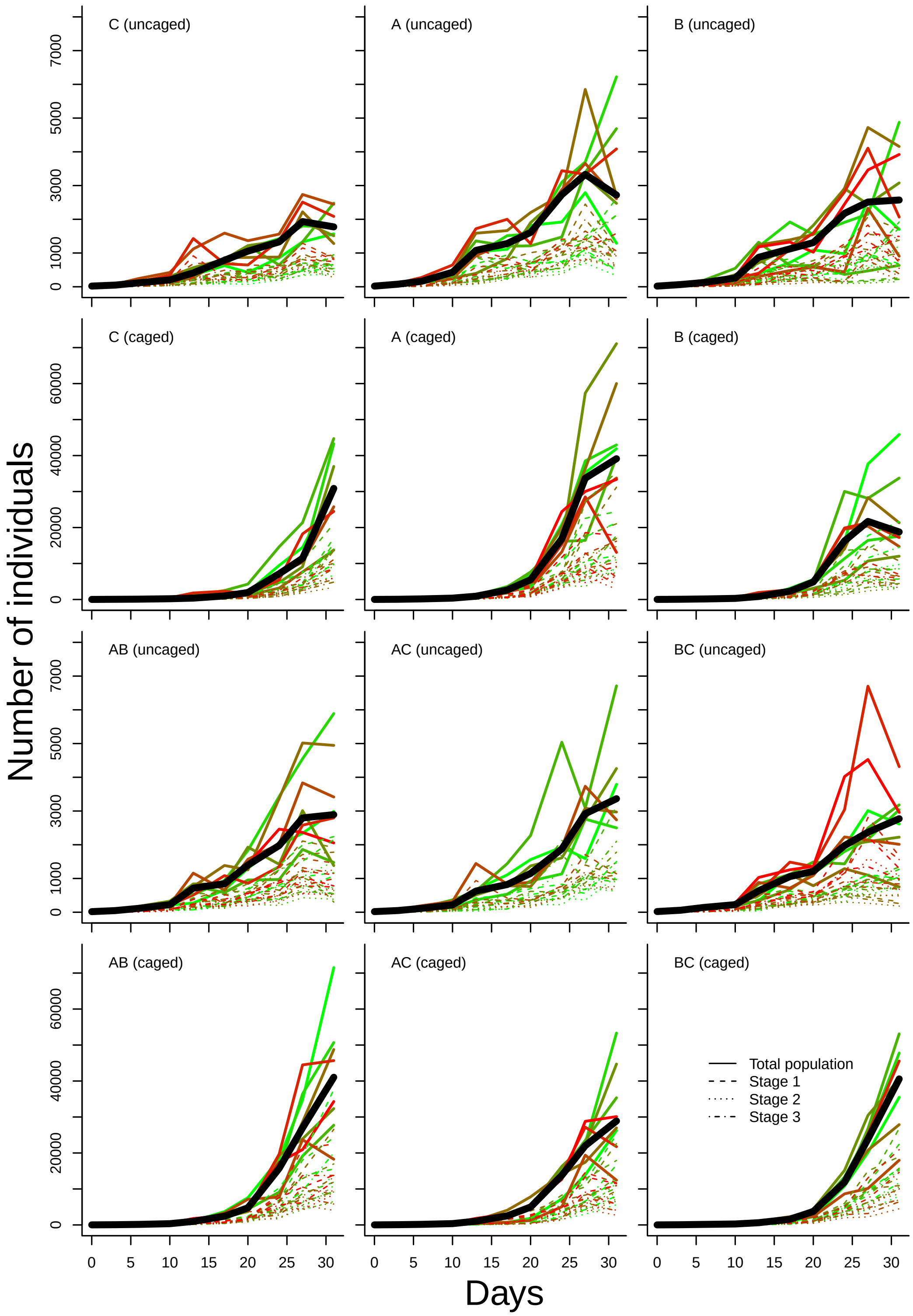

Figure S2: Changes in the individuals over time for each population, shown per experimental treatment. Solid lines show the total population sizes, different dotted lines show the numbers per developmental stage. Thick black line shows the median total population size per treatment. Different colors indicate different replicates, and correspond to the colors shown in Fig. S1. Note the different y-axis for the caged (row 2,4) and uncaged (row 1,3) treatment.

S1.3 Population densities

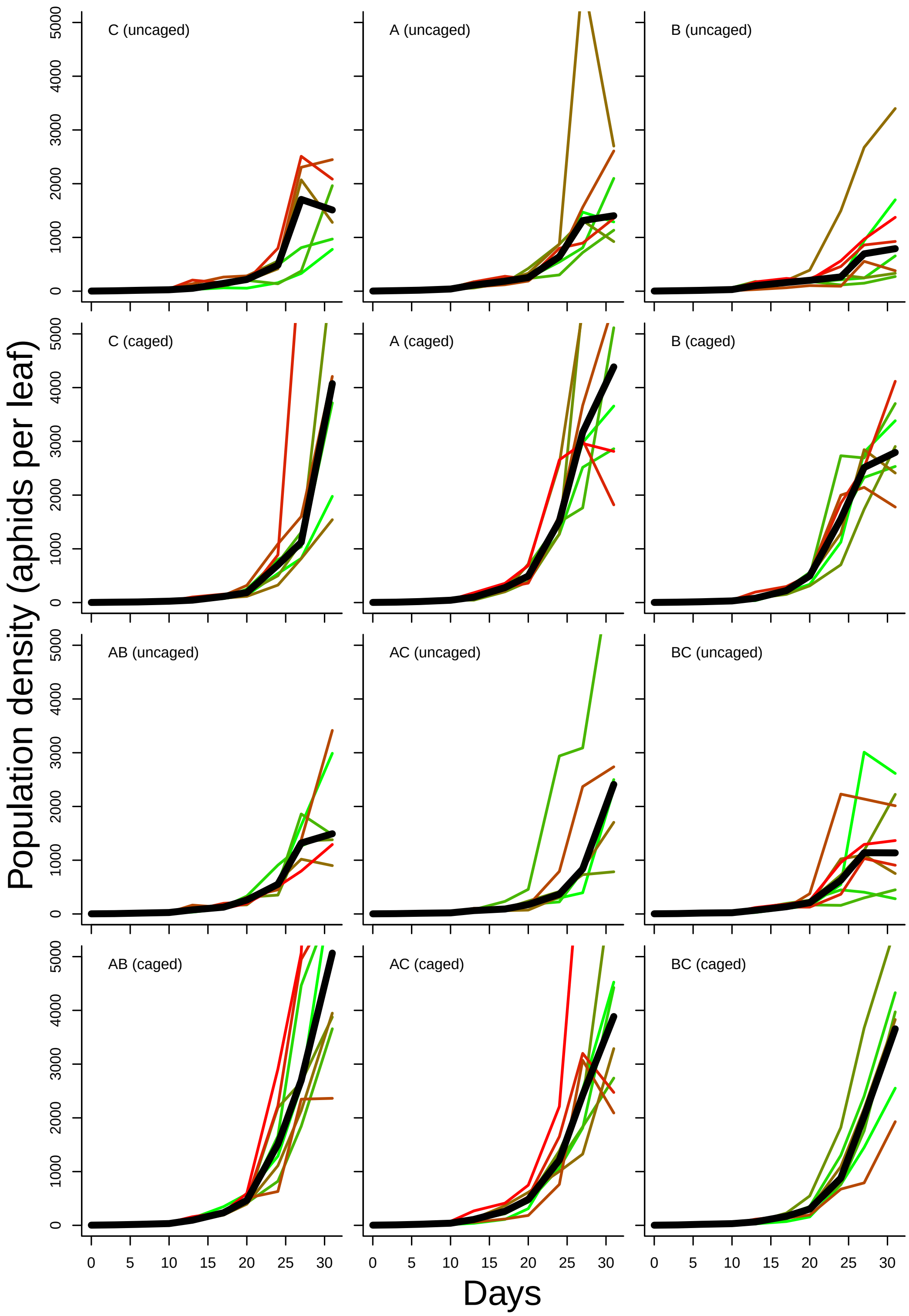

Figure S3: Changes in the densities (number of individuals per leaf) over time for each population, shown per aphid treatment. These results are obtained by dividing observed population numbers (Fig. S2) by the number of leaves (Fig. S1). Lines show the total population densities. Thick black line shows the median density per treatment. Different colors indicate different replicates, and correspond to the colors shown in Fig. S1.

S1.4 Clonal frequencies

We quantified which processes were most important in determining transient daily population growth rates of the evolving populations (results presented in Fig. 4 of the manuscript). To assess the importance of changes in clonal frequencies (evolution), we used the observed clonal frequencies. On day 13, 20 and 31, between 16 and 32 aphids from each plant were genotyped at three microsatellite loci (for more details, see Turcotte et al., 2011).

We performed a linear regression between frequency and day, for each aphid treatment separately (results below). Subsequently, the predicted frequencies at each measuring day, were used to calculate weighted vital rates of the evolving populations (see the manuscript for more details).

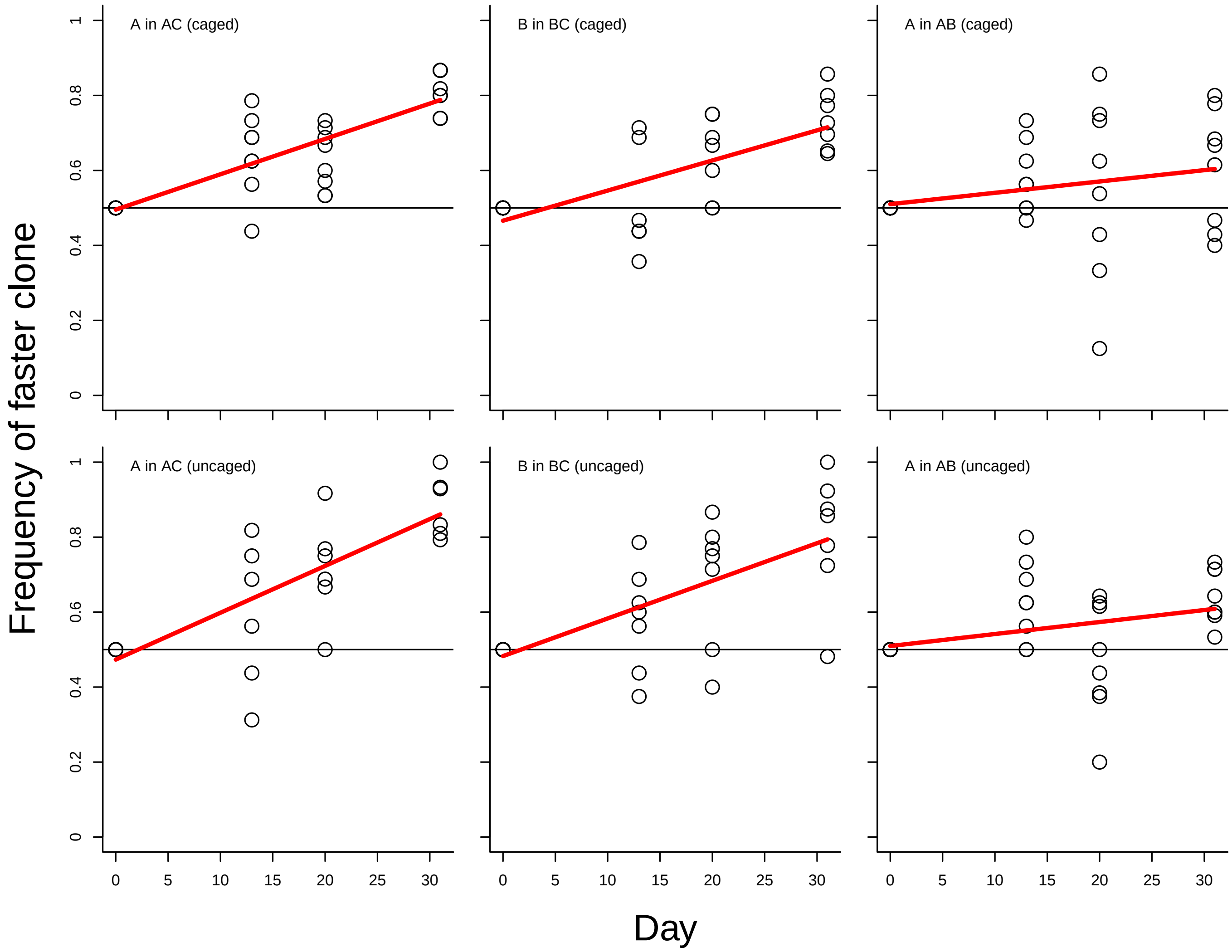

Figure S4: Frequency of the most frequent clone in each evolution treatment. Dots represent the observed frequencies for different replicates, and red lines indicate the fit based on a linear regression.

S2 Implementation in JAGS

Below the JAGS code of the final model is provided. Here, each vital rate was a function of density, clone and caging, with interactions between clone and caging.

```
model
{
  for (i in 1:N) {

    pred[1:3,i,1] <- obs[i,1:3]

    for (k in 1:4) { ## Project for 1-4 days

      ndens[i,k] <- (((sum(pred[1:3,i,k]) / plant[i,k]) - sfDens2[1]) / sfDens2[2])

      logit(s[i,k]) <- b0s + b7s1 * ndens[i,k] + b3s1[clone[i]] +
                       b2s1[clone[i]] * caging[i]
      logit(g[i,k]) <- b0g + b7g1 * ndens[i,k] + b3g1[clone[i]] +
                       b2g1[clone[i]] * caging[i]
      log(f[i,k]) <- b0f + b7f1 * ndens[i,k] + b3f1[clone[i]] +
                     b2f1[clone[i]] * caging[i]

      # fill matrix
      mat[1,1,i,k] <- s[i,k] * (1-g[i,k])
      mat[2,1,i,k] <- s[i,k] * g[i,k]
      mat[3,1,i,k] <- 0

      mat[1,2,i,k] <- 0
      mat[2,2,i,k] <- s[i,k] * (1-g[i,k])
      mat[3,2,i,k] <- s[i,k] * g[i,k]

      mat[1,3,i,k] <- f[i,k]
      mat[2,3,i,k] <- 0
      mat[3,3,i,k] <- s[i,k]

      pred[1:3,i,k+1] <- mat[1:3,1:3,i,k] %*% pred[1:3,i,k]
    }
  }

  # likelihoods
  for (i in rest) {
    summ[i] <- sum(pred[1:3,i,diffDays[i]+1]) + 0.003
    prob[i,1] <- (0.001+pred[1,i,diffDays[i]+1]) / summ[i]
    prob[i,2] <- (0.001+pred[2,i,diffDays[i]+1]) / summ[i]
    prob[i,3] <- (0.001+pred[3,i,diffDays[i]+1]) / summ[i]

    dens2[i,1:3] ~ dmulti(prob[i,1:3],densTot[i])
    densTot[i] ~ dpois(summ[i])
  }

  # save for cross validation
  for (i in cv) {
      crossval[1:3,i] <- pred[1:3,i,diffDays[i]+1]
  }

  ## Individual likelihoods
  logit(surv0) <- b0s + b7s1 * ((1-sfDens2[1]) / sfDens2[2]) # surv at density=1
```

```
  log(repr0) <- b0f + b7f1 * ((1-sfDens2[1]) / sfDens2[2]) # repr at density=1
  logit(growth0) <- b0g + b7g1 * ((1-sfDens2[1]) / sfDens2[2]) # growth at density=1
  mg <- 1 + 1/growth0 + 1/growth0 # average time to arrive at stage 3 (=mature)

  m <- 1-surv0
  for (i in 1:NindS) {
    survObs[i] ~ dexp(m)
  }
  for (i in 1:NindR) {
    reprObs[i] ~ dpois(repr0)
  }
  for (i in 1:NindG) {
    growthObs[i] ~ dgamma(sh,ra)
  }
  # mean (mg) and standard deviation (sd)
  sh <- pow(mg,2) / pow(sd,2)
  ra <- mg / pow(sd,2)
  sd ~ dunif(0,100)

  ## Priors
  # intercepts
  taub0s <- 1/(0.65*0.65) # approximately normal on logit scale
  b0s ~ dnorm(0,taub0s)
  b0g ~ dnorm(0,taub0s)
  b0f ~ dnorm(0,0.1)

  # Density effects
  b7s1 ~ dnorm(0,0.1)
  b7g1 ~ dnorm(0,0.1)
  b7f1 ~ dnorm(0,0.1)

  # Caging effects
  for (i in 1:6) {
    b2s1[i] ~ dnorm(0,0.1)
    b2g1[i] ~ dnorm(0,0.1)
    b2f1[i] ~ dnorm(0,0.1)
  }

  # Clone effects
  for (i in 1:6) {
    b3s1[i] ~ dnorm(0,0.1)
    b3g1[i] ~ dnorm(0,0.1)
    b3f1[i] ~ dnorm(0,0.1)
  }
}
```

S3 Model verification

We performed four analyses for model verification: i) We first tested seven models including different covariates, fitted to each aphid treatment separately, to select the covariate resulting in the best fit (Section S3.1). ii) We tested our inverse modelling approach with simulated data (Section S3.2). iii) We looked at the residuals of the fitted model (Section S3.3). Finally, iv) we reran the model six times testing a wide range of initial values. In these six runs, the medians of the posterior distributions were equal, giving confidence that a global optimum was found.

S3.1 Testing different covariates

In order to evaluate which covariate resulted in the best model fit, we started by testing seven different 'basic' models. These models were fitted for each of the six aphid treatments ('A', 'B', 'C', 'AB', 'AC', 'BC') separately to assess the performance of different modelling structures for each aphid treatment independently. These models were: a null model (including only a survival, growth and reproduction intercept), and six models including intercepts, together with additive effects of developmental stage enabling different 1) survival and 2) growth for different stages x, 3) caging (C), 4) population size (N), 5) plant size (P) and 6) population size divided by plant size to obtain densities (number of individuals leaf^{-1}; D). We refer to these models as Models 0-6 (see Table S1 for an overview). Caging was included as a factor with two levels (caged and uncaged). For the model including a caging effect, parameters for the uncaged treatments were estimated including data from day 14 and onwards only, since cages were removed at day 13. Observations until day 13 were used to estimate parameters for the caged treatments. Population size was included as a continuous variable, and we transformed values by Z-score normalization (using the mean and standard deviation calculated across the full dataset). We used the number of leaves as a proxy for plant size, included as a continuous covariate after Z-score normalization. Finally, densities were obtained by dividing population size by plant size, to obtain number of individuals per leaf, and values were normalized.

To assess the predictive ability of a fitted model in order to compare different models, we used K-fold cross-validation. Each basic model was fitted eight times, each time removing one replicate per caging treatment for validation. This implied that two populations were removed per fit (one from both caging treatments), as parameters were fitted per aphid treatment and each aphid treatment was exposed to both caging treatments. Model predictions were calculated for the validation data, and the cross-validation score was calculated as the mean squared error between the predictions and observations. The best model was considered the model resulting in the lowest overall mean error (i.e. the highest out-of-sample predictive ability), across aphid treatments.

As can be seen in Table S1, the model including density (D; model 6) resulted in the lowest mean error overall. Based on this, we constructed a full model including density, fitted across aphid treatments on the complete data set. As visual observations revealed an additional effect of caging after accounting for plant size (Fig. S3), we additionally included an effect of caging, estimated per aphid treatment (see Eq. 1 of the manuscript for the structure of the fitted model). Note that for this final model, we included two sources of data. First, population-level observations as obtained during the experiment, and second, individual life table data collecting during a greenhouse experiment (explained in more detail in the manuscript). To compare the seven models including different covariates, we only compared population-level predictions with observations, and did not include individual life table data. This was done in order to select the best covariate based on only the experimental data.

Table S1: Root of the mean squared error of out-of-sample prediction for different basic models, for each aphid treatment. Models include different predictors, and thereby differ in how the vital rates were calculated. Variable x is developmental stage, C is caging, N is population size, P is plant size and D is density. Predicted $\hat{y}$ is translated to daily survival (σ), growth (γ) and reproduction (ϕ) using the relevant link function. Model 6 resulted in the best model overall and was used as a basis for the full model.

		C	A	B	AB	AC	BC	Mean
Model 0	$\hat{y} = \beta_0$	1104	2273	1633	1899	1276	987	1529
Model 1	$\hat{y_s} = \beta_{0_s} + \beta_{1_s}x$	1102	2276	1634	1873	1252	974	1518
Model 2	$\hat{y_g} = \beta_{0_g} + \beta_{1_g}x$	1104	2283	1640	1886	1256	971	1523
Model 3	$\hat{y} = \beta_0 + \beta_1 C$	1136	2363	1678	1957	1271	1034	1573
Model 4	$\hat{y} = \beta_0 + \beta_1 N$	1120	1901	1634	1695	1357	960	1444
Model 5	$\hat{y} = \beta_0 + \beta_1 P$	930	2736	5771	1708	1327	894	2228
Model 6	$\hat{y} = \beta_0 + \beta_1 D$	982	1871	1111	1551	1252	864	**1272**

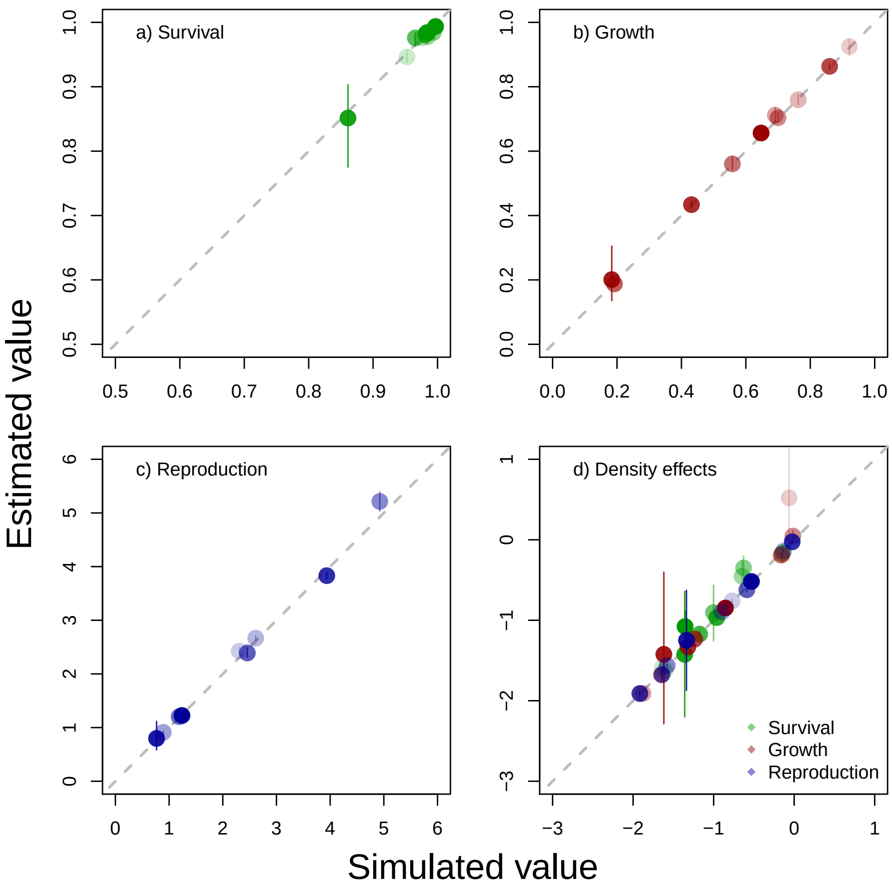

Figure S5: Simulated parameters against estimated parameters for a) survival, b) growth, c) reproduction and d) density dependence. Levels of transparency indicate different simulations. Error bars show 95% credible intervals. In d) different colors indicate density-effects on survival (green), growth (red) and reproduction (blue).

S3.2 Simulated data

To ensure that the defined model was identifiable and that the 'correct' parameters could be estimated, we tested our inverse modelling approach with simulated data (for which the true relationships are known). We simulated data according to the selected model 6 (Table S1), assuming a constant plant size (in case it equals model 4; see Table S1).

Random survival, growth and reproduction parameters were drawn, as well as density coefficients; the latter constrained to be below zero (in order to only simulated negative density dependence). Starting with 20 individuals in stage 2, we simulated (density-dependent) dynamics for 31 days. This was repeated three times to create three replicates. Per replicate, normally-distributed noise was added to each of the parameters (standard deviation 0.05), leading to some variation between replicates. We recorded population size and stage composition on the same days as in the original experiment (i.e. every three or four days). We then continued with the simulations whenever population size of all replicates at day 31 was between 634 and 71512 individuals, which was the range of observed population sizes at day 31, to include only those parameter combinations that were realistic in the context of this experiment. This was the case in ≈85% of the simulations.

Using these data, model 6 was fitted in the same way as the experimental data. We compared estimated parameters with the real, simulated, values. This was repeated ten times, each time with a different parameter set. Estimated parameters were close to the simulated parameters for the entire range of parameters and they were unbiased (Fig. S5).

S3.3 Residuals

For the uncaged populations, predictions were unbiased and accurate for the entire range of population sizes (Fig. S6). For the caged treatments, absolute error increased with population density and predictions were slightly biased at the highest densities.

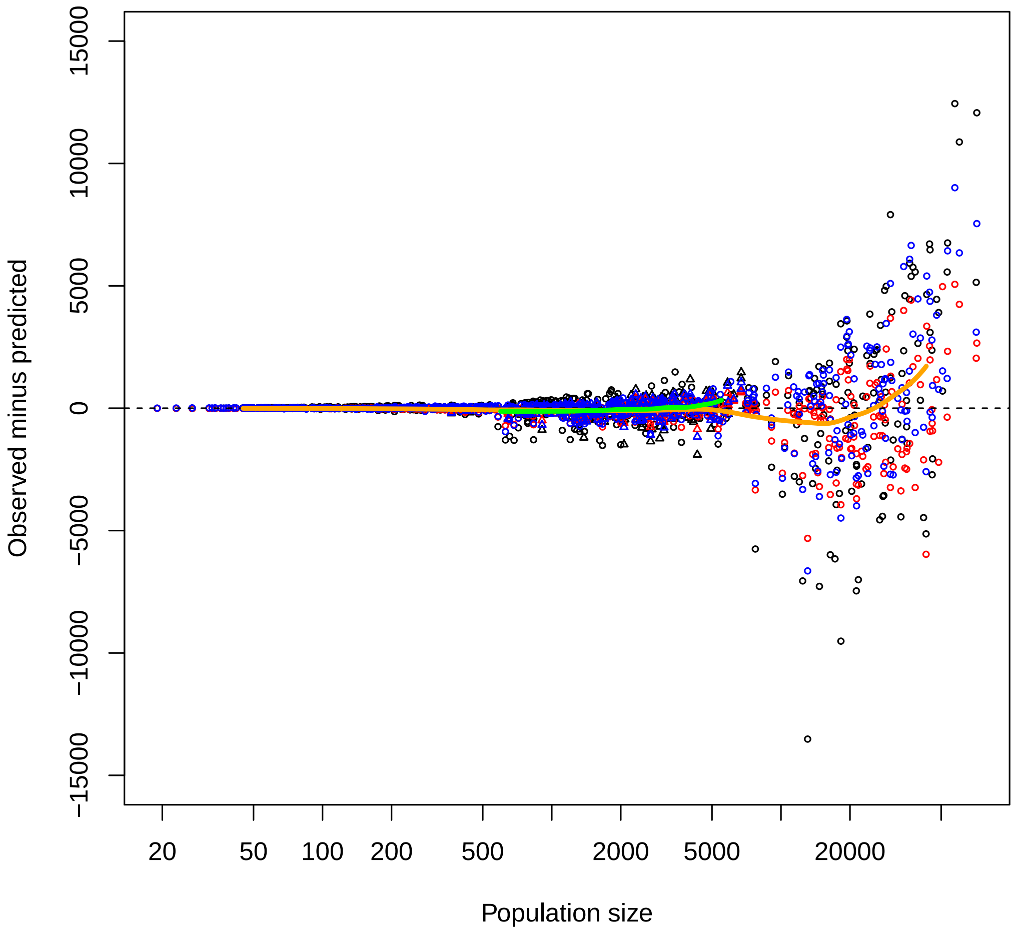

Figure S6: Observed minus predicted number of individuals over (log) population sizes. Different symbols show uncaged (triangles) and caged (circles) treatments, and different colors indicate different developmental stages (1: black, 2: red, 3: blue). Lines show moving average across all stages for the caged (orange) and uncaged (green) treatments, based on generalized additive models, excluding lower and upper 2.5 % of the population size data.

S4 Model results

S4.1 Posterior estimates

Table S2: Posterior estimates of the fitted model. Middle column gives the median values of the posterior distributions (which were used for the results presented in Figs 1-4 of the manuscript). Left and right columns give the lower and upper estimate of the 95% credible intervals. Parameters β_0 represent the intercepts, parameters β_1 represent density effects, clonal effects are indicated by subscripts and β_2 represent the interactions between clone and cage removal. Subscripts ϕ, γ and σ indicate parameters influencing reproduction, growth and survival, respectively.

	Lower95	Median	Upper95
$\beta_{0\sigma}$	4.03	4.28	4.54
$\beta_{0\gamma}$	-2.33	-2.29	-2.24
$\beta_{0\phi}$	0.23	0.28	0.34
$\beta_{1\sigma}$	3.68	3.72	3.77
$\beta_{1\gamma}$	-2.34	-2.31	-2.29
$\beta_{1\phi}$	-1.40	-1.39	-1.38
$\beta_{C\phi}$	0.06	0.11	0.17
$\beta_{A\phi}$	0.19	0.24	0.30
$\beta_{B\phi}$	-0.45	-0.40	-0.34
$\beta_{AB\phi}$	0.14	0.19	0.25
$\beta_{AC\phi}$	-0.06	-0.00	0.05
$\beta_{BC\phi}$	-0.10	-0.05	0.01
$\beta_{C\gamma}$	2.83	2.89	2.94
$\beta_{A\gamma}$	2.49	2.54	2.59
$\beta_{B\gamma}$	1.80	1.86	1.91
$\beta_{AB\gamma}$	2.51	2.57	2.62
$\beta_{AC\gamma}$	2.18	2.24	2.29
$\beta_{BC\gamma}$	2.40	2.45	2.51
$\beta_{C\sigma}$	-2.57	-2.32	-2.06
$\beta_{A\sigma}$	-2.49	-2.23	-1.98
$\beta_{B\sigma}$	-0.96	-0.70	-0.44
$\beta_{AB\sigma}$	-2.59	-2.33	-2.08
$\beta_{AC\sigma}$	-2.18	-1.92	-1.67
$\beta_{BC\sigma}$	-2.14	-1.89	-1.63
$\beta_{2C\phi}$	-2.19	-2.14	-2.09
$\beta_{2A\phi}$	-1.94	-1.91	-1.89
$\beta_{2B\phi}$	-1.64	-1.61	-1.57
$\beta_{2AB\phi}$	-1.87	-1.84	-1.82
$\beta_{2AC\phi}$	-1.11	-1.05	-1.00
$\beta_{2BC\phi}$	-1.83	-1.80	-1.77
$\beta_{2C\gamma}$	-3.07	-3.00	-2.93
$\beta_{2A\gamma}$	-2.29	-2.24	-2.19
$\beta_{2B\gamma}$	-2.06	-2.00	-1.94
$\beta_{2AB\gamma}$	-2.19	-2.14	-2.09
$\beta_{2AC\gamma}$	-1.10	-1.00	-0.91
$\beta_{2BC\gamma}$	-2.43	-2.38	-2.33
$\beta_{2C\sigma}$	4.52	6.64	9.61
$\beta_{2A\sigma}$	5.48	7.45	10.29
$\beta_{2B\sigma}$	4.38	6.53	9.53
$\beta_{2AB\sigma}$	6.30	8.29	10.88
$\beta_{2AC\sigma}$	0.82	0.98	1.15
$\beta_{2BC\sigma}$	5.83	7.79	10.58

S4.2 LTRE for uncaged treatments

The analyses presented in Fig. 2 of the manuscript were performed for the caged treatments. Below we show the results when done for the uncaged treatments.

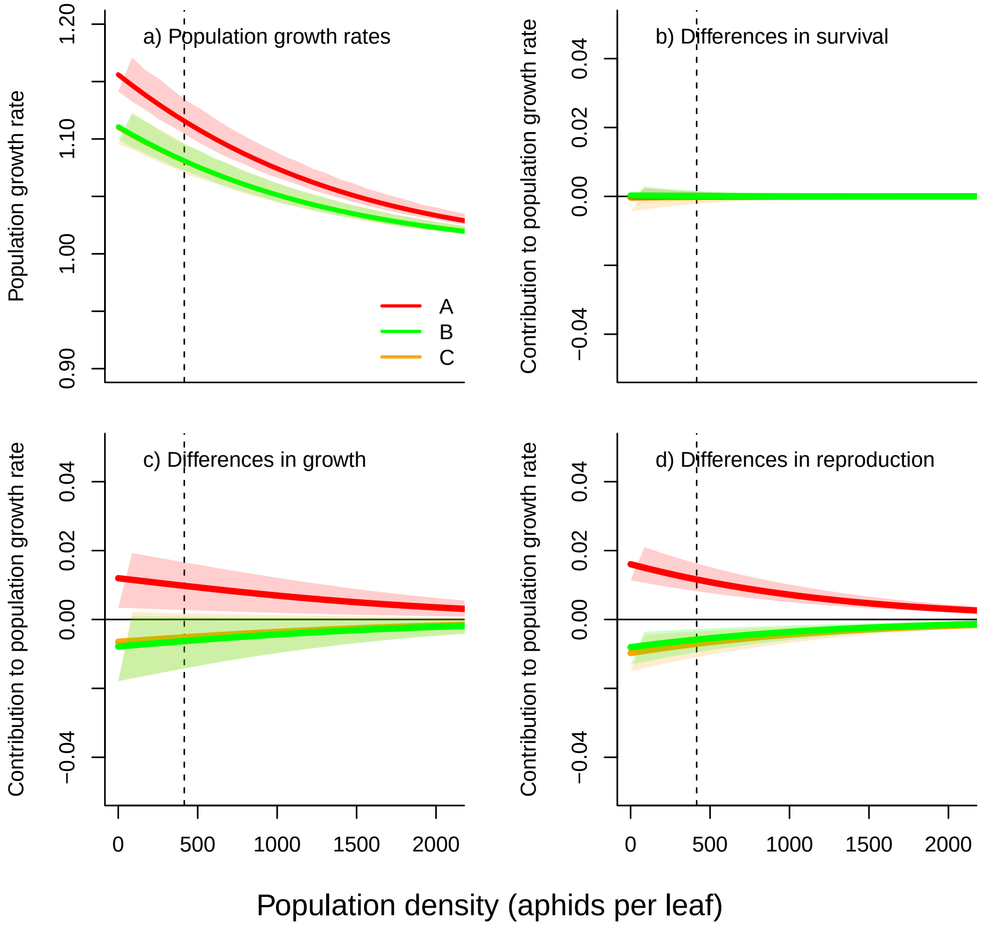

Figure S7: a) Projected population growth rate as a function of density for the three pure clones, under uncaged conditions. Population growth rate of clone C was essentially the same as population growth rate of clone B, and is therefore not visible. b-d) Life Table Response Experiment comparing different clones, as a function of density (individuals per leaf). Clone A-C were compared to the average matrix across the three clones. Densities range between 0 and the 95% quantile of observed densities, under uncaged conditions. Different colors indicate different clones and vertical lines indicate the average density under uncaged conditions. Shaded polygons show 95% confidence intervals in the predictions, obtained by simulating 1000 transition matrices by drawing coefficients from the posterior distributions of the clonal effects.
